# Ball Mill Beatdown: Mechanochemical Synthesis of TaON Nanocrystals

**DOI:** 10.1021/acs.inorgchem.6c00957

**Published:** 2026-05-15

**Authors:** Eve K. Stegner, Madison R. Kuns, Kerly Ochoa-Romero, Gonzalo Guirado, Javier Vela

**Affiliations:** † Department of Chemistry, 1177 Iowa State University, Ames, Iowa 50011, United States; ‡ Departament de Química, 16719 Universitat Autonòma de Barcelona, Cerdanyola del Vallès, 08193 Barcelona, Spain; § US DOE Ames National Laboratory, Ames, Iowa 50011, United States

## Abstract

Semiconductor photocatalysts play a key role in advancing sustainable fuel production, air purification, and water treatment. The rapid growth of heterogeneous photocatalysis is further supported by the development of flow-based photoreactors, which have paved the way toward pilot- and commercial-scale photocatalysis. However, their operation often relies on stable suspensions of the catalyst in solution, posing a significant barrier to entry for many promising photocatalysts. Specifically, oxynitride semiconductors, some of the leading contenders in photochemical water splitting, tend to undergo rapid sedimentation. To address this challenge, we investigate mechanochemical methods of synthesizing dispersible TaON nanocrystals. We show that it is possible to prepare <20 nm TaON nanocrystals from both pre- and postnitridation ball milling, with the latter method enabling particle size control. TaON nanocrystals prepared by both methods form significantly more stable suspensions than do their bulk counterparts. We demonstrate that TaON nanocrystals prepared by both routes display similar photocatalytic activity to their bulk forms while exhibiting remarkable stability under neutral and acidic conditions. Further, we demonstrate that Ag and C electrodes modified with TaON nanocrystals achieve a substantial decrease of more than 0.8 V in cathodic peak potentials for CO_2_ electroreduction. These combined results indicate that ball milling produces stable, dispersible, and catalytically active oxynitride nanocrystals, extending their potential applications from laboratory to large-scale catalysis.

## Graphical abstract

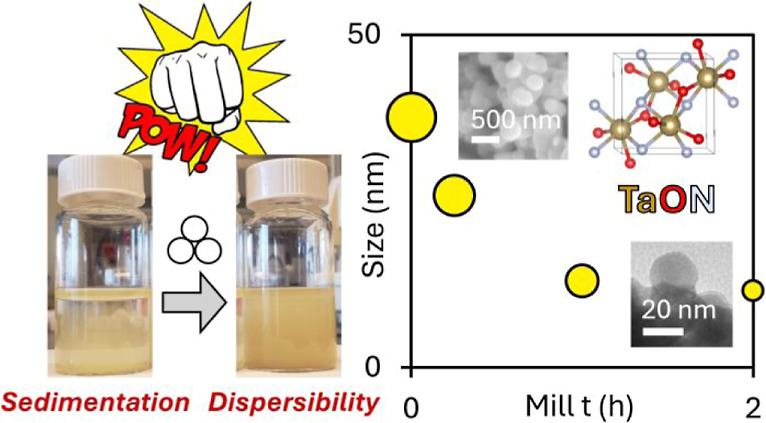

## Introduction

Photo- and electrocatalysts are responsible for facilitating important chemical transformations using sunlight, a cheap, clean, and abundant energy source.^1–6^ TaON and Ta_3_N_5_ are among the most promising catalysts, initially gaining notoriety as excellent candidates for overall water splitting.^7–15^ TaON and Ta_3_N_5_ have numerous advantages in comparison to more ubiquitous alternatives such as TiO_2_. For instance, they exhibit visible light absorption (λ > 380 nm) and have suitable band positions for hydrogen production,^16,17^ carbon dioxide reduction,^18–20^ and nitrate reduction (Figure 1).^21^ The rise of TaON and Ta_3_N_5_ further spearheaded the synthetic development of less-studied perovskite oxynitrides such as AeTaO_2_N (Ae = Mg, Ca, Sr, Ba), which also display promising photo- and electrocatalytic activity.^22–28^ While these catalysts are suitable for laboratory scale reactions, scaling up these materials to pilot scale and beyond introduces additional challenges.

**Figure 1:**
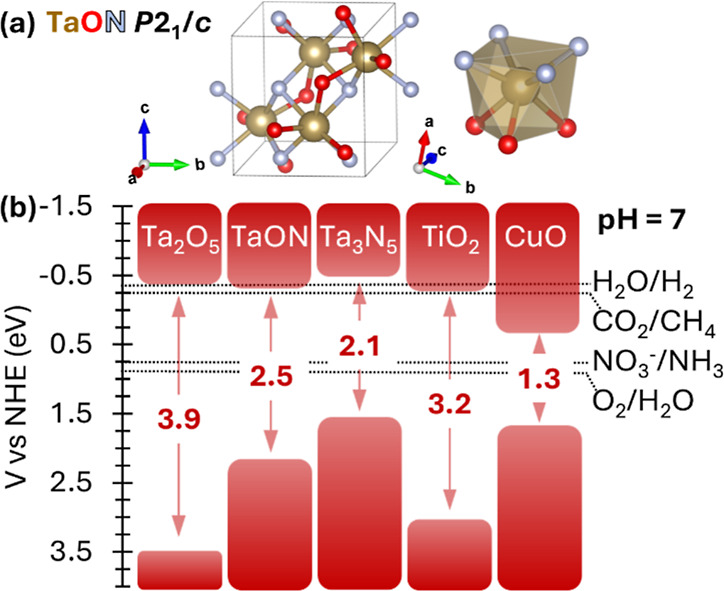
(a) Unit cell structure of TaON and local coordination environment of Ta. (b) Band positions of selected semiconductors with respect to the reduction potentials of relevant redox reactions (TiO_2_ anatase; band gaps labeled in red).

Currently, pilot scale and larger photocatalysis applications employ multichannel flow reactors,^29,30^ which circulate a solution of reagents and catalyst dispersed through a series of small, transparent tubes, maximizing the area exposed to sunlight. To prevent sedimentation inside the reactor, it is imperative that the catalyst and solvent form a stable suspension.^31–34^ Unfortunately, numerous photocatalysts, such as bulk oxynitrides, currently lack the colloidal stability required for such applications. Thus, it is desirable to develop dispersible, nanoscale catalysts to meet the demands of existing large-scale flow reactors and infrastructure.^35–38^

To date, reports on nanocrystalline oxynitrides remain limited.^39–41^ Nitrides and oxynitrides are commonly prepared by high-temperature nitridation, in which ammonia (NH_3_) gas passes over a mixture of metal oxides and/or chlorides at temperatures exceeding 800 °C for 1–24 h or longer.^11,13,27^ High-temperature nitridation can be difficult to adapt to colloidal nanoscale synthesis, as the only tunable parameters in nitridation are time, temperature, and flow rate, all of which tend to affect nitrogen content rather than the particle size.^24,27^ On the other hand, bottom-up synthetic approaches toward colloidal oxynitride materials remain limited, with initial preparations resulting in air-sensitive Ta_3_N_5_ nanocrystals.^39^ The scarcity of nanoscale-specific preparations justifies the need to explore alternative pathways toward dispersible oxynitrides.

Ball milling, commonly utilized in chemical synthesis,^42–45^ catalysis,^43,46^ and materials processing,^45^ has gained considerable attention as a scalable method for the preparation of semiconductor nanocrystals from their bulk forms.^47–51^ In a recent example, our group reported that physical and mechanical methods of size reduction such as hand-grinding and ball milling can reduce the particle size while also increasing catalytic activity.^52^ Furthermore, mechanochemistry can circumvent the need for potentially hazardous or wasteful solvents and surfactants generally required in the bottom-up synthesis and purification of colloidal nanocrystals.^46,53^ Here, we examine ball milling as a versatile synthetic tool to access dispersible TaON nanocrystals. Ball milling either prior to or after nitridation produces nanocrystals which form significantly more stable suspensions compared to bulk TaON. The new nanocrystals show promising photo- and electrocatalytic activity and stability. For example, we show that TaON-modified electrodes display improved behavior toward the electrochemical reduction of CO_2_, a reaction of high relevance to sustainable carbon-management technologies due to its potential to turn greenhouse gas emissions into fuels and value-added chemicals.^54,55^

## Results and Discussion

### TaON Nanocrystals via Prenitridation Ball Milling

The partial nitridation of Ta_2_O_5_ to TaON is traditionally considered to be a pseudomorphous exchange between oxygen and nitrogen.^56^ Hence, the particle size and morphology tend to remain relatively similar prior to and after nitridation. Therefore, to reduce the particle size of TaON, we first considered reducing the particle size of the precursor, Ta_2_O_5_, through ball milling. Commercially available Ta_2_O_5_ powder is poorly dispersible, settling out of solution in as little as 1 min. Solutions prepared by briefly suspending bulk Ta_2_O_5_ in ultrahigh purity water and passing it through a 100 nm syringe filter fail to provide a signal in dynamic light scattering (DLS) experiments. Scanning electron microscopy (SEM) images of bulk Ta_2_O_5_ reveal relatively large average particle sizes of 380 ± 90 nm (Figure 2). However, because the single crystalline domain (Scherrer) sizes—obtained from analysis of peak broadening in the powder X-ray diffraction (XRD) pattern—are much smaller, at about 27 ± 14 nm (Figure 3 and Table 1), we conclude that each particle in bulk Ta_2_O_5_ is made of numerous, highly twinned, smaller crystals. These results confirm the presence of large (>100 nm) particles in the commercial Ta_2_O_5_ powder and underscore the incentive to reduce particle size.

**Figure 2:**
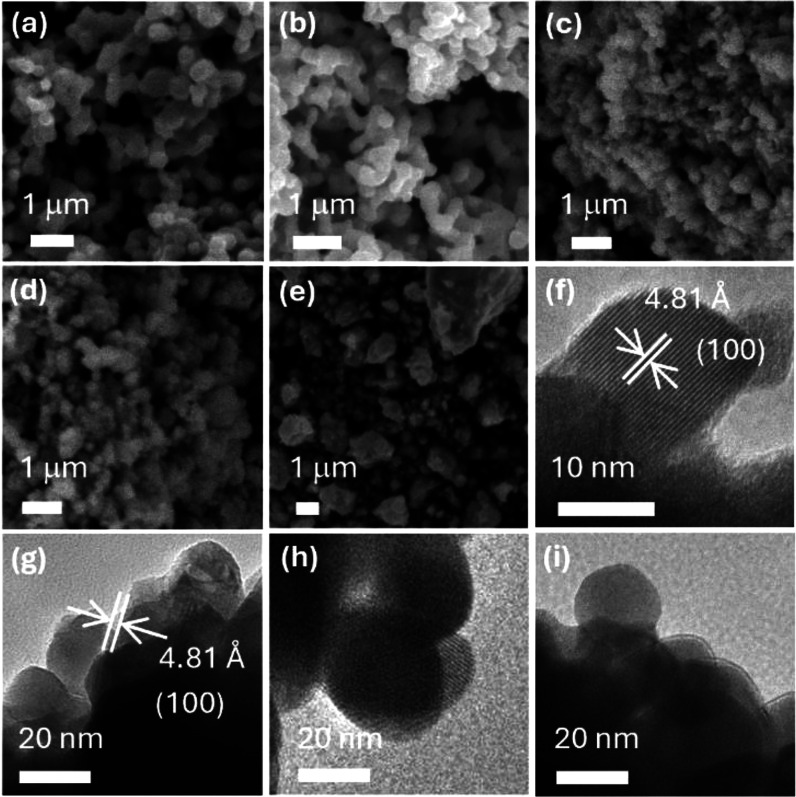
SEM/TEM images of (a) bulk Ta_2_O_5_, (b) bulk TaON, (c) ball-milled Ta_2_O_5_, and TaON prepared by (d) nitridation of ball-milled Ta_2_O_5_, or (e–i) ball milling bulk TaON.

**Figure 3:**
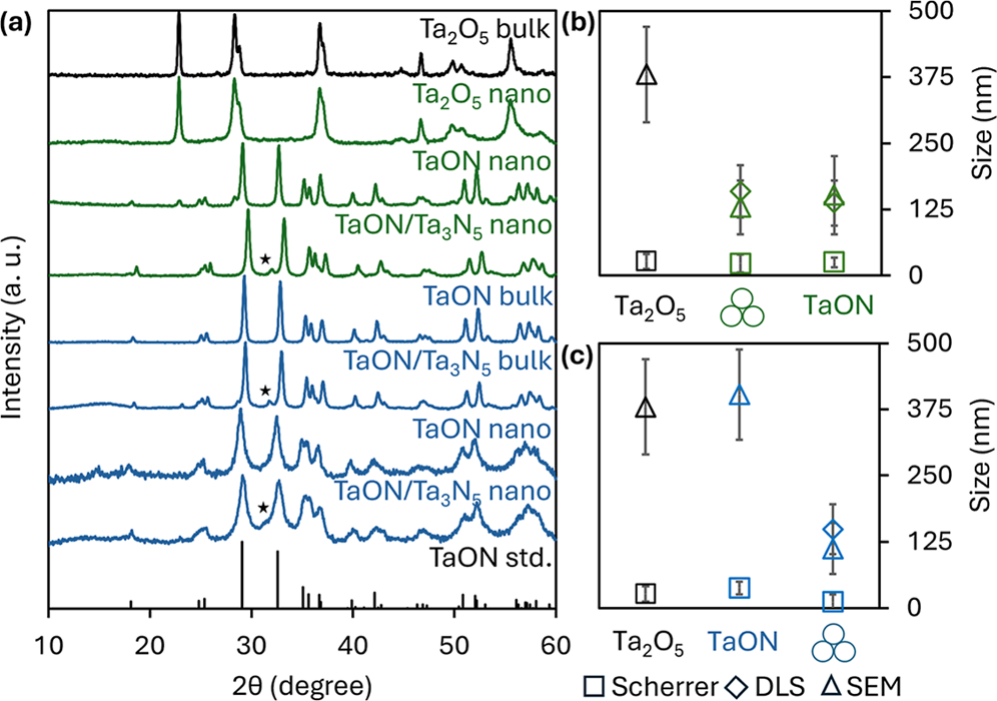
Powder XRD (a) of Ta_2_O_5_, TaON, and TaON/Ta_3_N_5_ (Ta_3_N_5_ reflection (023) indicated with a black star) nanocrystals along with resulting particle sizes (b,c) from prenitridation ball milling (green) and postnitridation ball milling (blue) (Ta_2_O_5_ *P*2*mm* #9112; TaON *P*2_1_/*c* #1032; Ta_3_N_5_ *Cmcm* #66533).

**Table 1:** Synthesis of TaON Nanocrystals

starting material	conditions	product	Scherrer size (nm)	SEM size [TEM] (nm)	DLS size (nm)	band gap (eV)	zeta potential (mV)
Ta_2_O_5_	commercial	NA	27 ± 14	380 ± 90	n.d.	3.9	–48 ± 9
Ta_2_O_5_ (bulk)	ball mill,^b^ RT, 2 h	Ta_2_O_5_ (nano)	23 ± 17	129 ± 51	159 ± 49	4.0	–40 ± 7
Ta_2_O_5_ (nano)	NH_3_,^a^ 850 °C, 3 h	TaON (nano)	25 ± 9	152 ± 77	137 ± 43	2.5	–36 ± 7
Ta_2_O_5_ (bulk)	NH_3_,^a^ 850 °C, 3 h	TaON (bulk)	38 ± 12	403 ± 85	n.d.	2.5	–44 ± 11
TaON	ball mill,^b^ RT, 0.25 h	TaON (nano)	26 ± 14	n.d.	n.d.	2.5	n.d.
TaON	ball mill,^b^ RT, 1 h	TaON (nano)	13 ± 10	n.d.	149 ± 47	2.5	n.d.
TaON	ball mill,^b^ RT, 2 h	TaON (nano)	12 ± 10	112 ± 48 [*20 ± 8*]	n.d.	2.5	–34 ± 7
TaON	ball mill,^b^ RT, 4 h	TaON (nano)	22 ± 11	N.d.	146 ± 38	2.5	n.d.

a 1 atm, 20 mL/min.

b The ball-to-powder ratio (BPR) was 9.1 (Ta_2_O_5_) or 12.7 (TaON).

Some discrepancies arise among particle sizes estimated through Scherrer analysis, electron microscopy, and DLS. For instance, Scherrer analysis estimates the crystallite size from the size-dependent peak broadening in powder X-ray diffraction patterns. It is common for individual particles to contain multiple, distinct crystalline domains separated by stacking faults or interspersed with amorphous regions.^57^ Consequently, Scherrer sizes are generally smaller than particle sizes determined by other techniques. In contrast, SEM provides overall measurements of the particle morphology and overall dimensions. Further, limited SEM resolution below approximately 100 nm can obscure particle boundaries, causing closely associated particles to appear as a single larger particle. This effect can lead to an overestimation of particle size in SEM images. DLS, on the other hand, reveals the hydrodynamic size of dispersed species in solution. Because the technique is highly sensitive to scattering from larger particles, even modest aggregation can dominate the DLS signal. As a result, DLS reports the size of particle aggregates rather than that of individual nanoparticles, frequently resulting in values larger than those obtained from structural or imaging techniques.

SEM imaging reveals that ball milling Ta_2_O_5_ for 2 h decreases average particle size from 380 ± 90 nm to 129 ± 51 nm (Scheme 1 and Figure 2). Further, powder XRD patterns of ball-milled Ta_2_O_5_ display a slight broadening of individual reflections, a characteristic of size reduction (Figure 3). Calculated Scherrer sizes indicate that ball milling Ta_2_O_5_ reduces the average single crystallite size from 27 ± 14 nm to 23 ± 17 nm (Table 1). In contrast to bulk Ta_2_O_5_, ball-milled Ta_2_O_5_ provides a strong DLS signal, with average hydrodynamic radii of 159 ± 49 nm. These findings indicate that ball-milled Ta_2_O_5_ particles are small enough to pass through a 100 nm filter, although these particles tend to amass together to form clusters of larger aggregates in solution. This result is consistent with the average particle size observed under SEM. Nevertheless, the ball-milled Ta_2_O_5_ nanocrystals form considerably more stable suspensions than the commercially available (bulk) Ta_2_O_5_ powder. Ta_2_O_5_ nanocrystals resist sedimentation for over 1 h, while bulk Ta_2_O_5_ undergoes clear sedimentation in as little as 1 min—see Figure S4 in the Supporting Information. This illustrates the effectiveness of ball milling in reducing particle size and enabling the formation of stable suspensions.

**Scheme 1:**
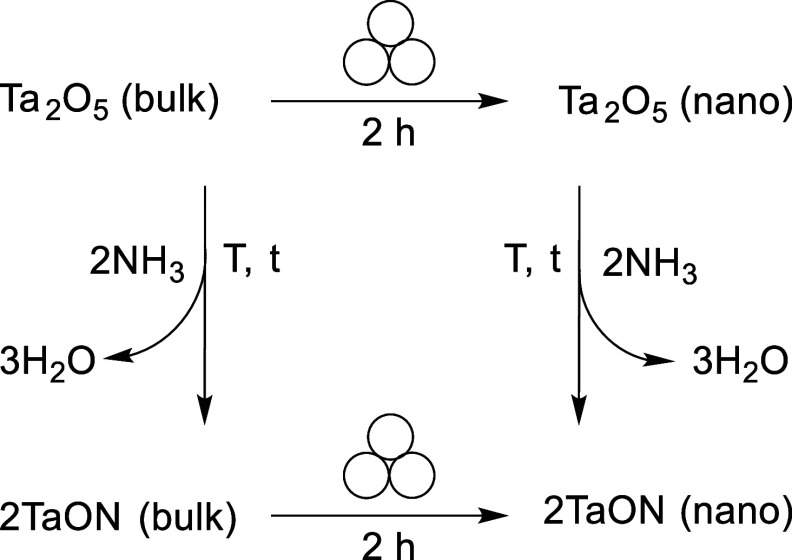
Synthesis of TaON Nanocrystals

Nitridation of Ta_2_O_5_ nanocrystals to TaON slightly increases particle size from 129 ± 51 nm to 152 ± 77 nm, as determined from SEM (Figure 2). SEM imaging also shows that both the precursor Ta_2_O_5_ nanocrystals and the resulting TaON nanocrystals exhibit spheroidal morphologies. These similar sizes and shapes are consistent with pseudomorphous exchange between oxygen and nitrogen during nitridation.^56^ Powder XRD reveals similar single crystallite (Scherrer) sizes prior to and post nitridation of 23 ± 17 nm and 25 ± 9 nm, respectively (Figure 3). Furthermore, DLS results show that particle size distributions remain consistent prior to and after nitridation. Specifically, upon nitridation, the average hydrodynamic diameter decreases slightly from 159 ± 49 nm to 137 ± 43 nm, confirming that dispersible TaON nanocrystals can be prepared from ball-milled Ta_2_O_5_ nanocrystals. Remarkably, TaON nanocrystals exhibit much slower sedimentation than bulk TaON prepared directly from bulk Ta_2_O_5_. TaON nanocrystals prepared from ball milled Ta_2_O_5_ remain suspended in aqueous solution for over 1 h, while bulk TaON undergoes rapid sedimentation in as little as 1 min (Figure S4). Ultimately, ball milling Ta_2_O_5_ proves to be an effective presynthetic modification for making TaON nanocrystals with outstanding dispersibility.

### TaON Nanocrystals via Postnitridation Ball Milling

Nitridation of bulk Ta_2_O_5_ with NH_3_ gas at 850 °C for 3 h forms phase pure, bulk TaON (Scheme 1 and Figure 3). SEM images reveal that nitridation of Ta_2_O_5_ to TaON slightly increases particle size from 380 ± 90 nm to 403 ± 85 nm, respectively (Figure 2). Because the average single crystallite (Scherrer) size falls within the nanoregime at 38 ± 12 nm, we conclude that each particle in bulk TaON is also made of numerous, highly twinned, smaller crystals, as discussed above (Figure 3). DLS confirms that the hydrodynamic particle size of bulk TaON remains above 100 nm, explaining the difficulty in forming stable suspensions of this material.

Ball milling the as-prepared bulk TaON for 2 h decreases particle size from 403 ± 85 nm to 112 ± 48 nm, according to SEM (Scheme 1 and Figure 2). Powder XRD results show that the single crystallite (Scherrer) size also decreases significantly, from 38 ± 12 nm to 12 ± 10 nm. DLS results confirm that ball milling TaON decreases particle size. For instance, DLS measurements indicate that 1 h ball milling produces TaON nanocrystals capable of passing through the 100 nm filter with an average hydrodynamic diameter to 149 ± 47 nm. Thus, SEM and DLS results demonstrate that TaON nanocrystals tend to aggregate together. To determine the actual average particle size of isolated TaON nanocrystals, we resorted to high-resolution transmission electron microscopy (TEM). TEM imaging of ball-milled TaON nanocrystals show that individual crystallites are roughly spheroidal in shape with an average diameter of 20 ± 8 nm, similar to the single crystallite size determined by Scherrer analysis (Figure 2). These nanocrystals also show superior stability in solution in comparison to bulk TaON. Sonicated suspensions of ball-milled TaON remain suspended for over 1 h, while bulk TaON begins to crash out of solution after only 1 min.

Interestingly, postnitridation ball milling may also offer opportunities for particle size manipulation. As milling time increases, resulting powder XRD patterns display characteristic broadening in each of the reflections, suggesting a progressive decrease in the crystallite size (Figure S5). Between 0, 0.5, 1, 2, and 4 h of ball milling time, Scherrer sizes decrease from 38 ± 12 to 26 ± 14, 13 ± 10, 12 ± 10, and 22 ± 11 nm, respectively. The steepest and most notable change in the average Scherrer size, ∼50%, occurs between 0.5 and 1 h, decreasing from 26 ± 14 nm to 13 ± 10 nm, respectively. The apparent increase in the Scherrer size from 12 ± 10 to 22 ± 11 nm is likely a result of uncertainty in measurement, with the two sizes falling within standard deviation of one another. Further, DLS results show negligible difference between TaON ball milled for 1 and 4 h, with average hydrodynamic diameters of 149 ± 47 nm and 146 ± 38 nm, respectively. This suggests that ball milling time, and resulting average crystallite size, have little effect on formation and average size of TaON nanocrystal aggregates in solution. Nonetheless, examining the effect of milling time on particle size uncovers a substantial reduction in dimensionality within as little as 1 h of ball milling, which dramatically increases the dispersibility of TaON.

### Effects of Ball Milling

As milling time of TaON increases from 0 to 4 h, the resulting nanocrystals become noticeably darker. This difference in color suggests either the introduction of impurities or structural defects upon ball milling TaON. Careful examination of the powder XRD patterns and energy-dispersive spectroscopy (EDS) results out rules the presence of any crystalline or amorphous chemical impurities responsible for the stark color change (Figures 3 and S6). Diffuse reflectance spectra reveals that the absorption edge and corresponding band gap of each ball-milled TaON sample remains at its expected value of 2.5 eV, as determined by the Tauc method (Figure S8). Furthermore, the sizes of each nanocrystal (>10 nm) remain above the calculated Bohr radius for TaON (5.7 nm).^58^ This confirms that the nanocrystals are too large to exhibit quantum confinement effects. However, as ball milling time increases, the absorption edge of TaON appears to broaden, accompanied by the gradual darkening of color. This suggests the presence of lower energy, defect states, likely indicative of surface trap sites. It is therefore likely that the stark color change is a result of surface defects introduced from the harsh, milling process.^47,49,59–62^

In contrast, ball-milled Ta_2_O_5_ particles fail to show a similar, broadening characteristic in their absorption spectrum. This suggests that TaON is more susceptible to surface deformation than Ta_2_O_5_ under similar ball-milling conditions. In consequence, TaON nanocrystals prepared by prenitridation ball milling share a similar reflectance spectrum to bulk TaON. Both exhibit a steep absorption edge with a corresponding band gap of approximately 2.5 eV. Thus, each synthetic pathway, pre- and postnitridation ball milling, forms TaON nanoparticles with differing optical and surface characteristics. The characterization of possible surface defects and implications of these results is currently under study and will be reported in a future work.

### Photocatalytic Activity and Chemical Stability

It is clear from optical spectroscopy along with general appearance that TaON nanocrystals prepared by pre- and postnitridation ball milling display different surface characteristics. To preliminarily gauge the effect of these differences on photocatalytic activity of TaON, we degraded bromothymol blue in the presence bulk TaON, TaON nanocrystals prepared by prenitridation ball milling, and TaON nanocrystals prepared by postnitridation ball milling. Each photocatalyst was modified with a CuO cocatalyst and compared against P-25 TiO_2_, a commercially available photocatalyst with proven pilot scale water splitting utility. Hand-grinding CuO nanocrystals with each semiconductor facilitates the formation of heterostructured photocatalysts. These interfaces are stabilized in solution by electrostatic attraction between the semiconductor surface, with a negative zeta potential of ca. −20 to −40 mV, and the surface of the CuO modifier, with a positive zeta potential of +28 ± 6 mV (Table 1).^63^

Irradiation of a bromothymol blue solution under 420 nm light in the absence of photocatalysts causes 15% degradation after 1 h (Figure 4). In the presence of TiO_2_/CuO, bromothymol blue degrades by 23% under the same conditions. Interestingly, bulk TaON/CuO and TaON/CuO nanocrystals prepared from pre- and postnitridation ball milling display little activity in bromothymol blue degradation. However, introduction of a slight (14–20%) Ta_3_N_5_ impurity by partial overnitridation (see the Experimental Section) significantly improves activity in the resulting heterostructures. For instance, bulk Ta_3_N_5_/TaON/CuO degrades bromothymol blue by 50% in 1 h. Ball-milled Ta_3_N_5_/TaON/CuO results in similar dye degradation of 49%. Further, Ta_3_N_5_/TaON/CuO nanocrystals prepared by prenitridation ball milling show comparable activity with 52% degradation. The stark difference in photocatalytic activity between TaON and TaON/Ta_3_N_5_ can likely be attributed to the increased charge separation in the heterostructure, reducing charge carrier recombination.^3,64^ Similar effects have been observed in CaTaO_2_N/Ta_3_N_5_ in photocatalytic water splitting.^24,65^ Ultimately, these results signify that Ta_3_N_5_/TaON nanocrystals prepared by both pre- and postnitridation ball milling maintain the catalytic activity of their bulk forms.

**Figure 4:**
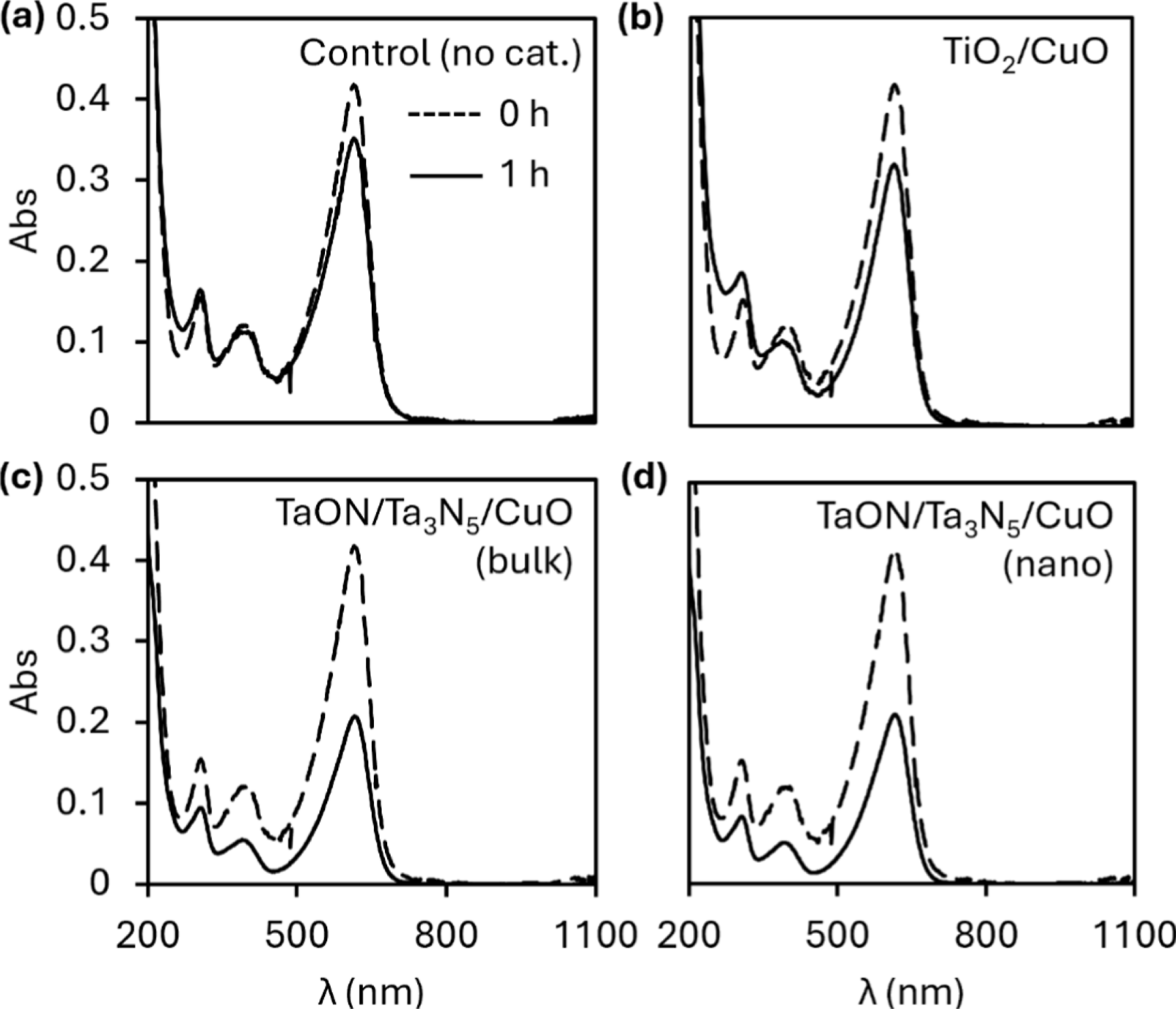
Photocatalytic degradation of bromothymol blue in the absence (a) of a catalyst, and in the presence of TiO_2_/CuO (b), TaON/Ta_3_N_5_/CuO (bulk) (c), and TaON/Ta_3_N_5_/CuO (nano) (d).

A key advantage of oxynitride semiconductors is their aforementioned stability under ambient conditions and in an aqueous solution. To confirm that these properties remained after ball milling bulk TaON, we tested the stability of ball-milled TaON nanoparticles with respect to aqueous solutions of pH 5 and 1. TaON nanocrystals were suspended in both ultrapure deionized water and 0.1 M HCl under continuous stirring for 96 h. Powder XRD results of the exposed TaON nanocrystals showcase outstanding resilience against oxidation, decomposition, and etching from exposure to both solutions. This indicates that these nanocrystals, despite their small sizes, retain the impressive stability of their bulk form. Furthermore, the stability of TaON nanocrystals in various pH environments indicates that they may be suitable catalysts for a wide range of pH-dependent catalytic transformations.

### Electroreduction of CO_2_ Using TaON-Modified Electrodes

CO_2_ electroreduction is often limited by poor kinetics, high overpotentials, and low selectivity. Therefore, identifying new catalytic materials for this reaction is essential.^66^ The aforementioned stability of TaON nanocrystals against etching in acidic solution inspired us to examine its preliminary electrochemical behavior in the context of CO_2_ reduction. TaON offers notable advantages in electrocatalysis including mixed ionic–electronic conductivity and stable catalytic surfaces—properties that make them strong candidates for future CO_2_ conversion applications.^25,58^ While tantalum nitride and oxynitride are active in hydrogen evolution (HER), oxygen evolution (OER), and oxygen reduction reactions (ORR),^67,68^ their application to electrochemical CO_2_ reduction (CO_2_RR) remains underexplored. Here, we used cyclic voltammetry (CV) to examine the influence of TaON as a modifier for screen-printed Ag and C electrodes against CO_2_ electroreduction. Notably, the dispersibility of TaON nanocrystals prepared by ball milling enables straightforward electrode modification via drop-casting, which can be performed at room temperature under standard atmospheric conditions. To avoid competing HER in water, we performed these reactions in an ionic liquid which—compared to other types of electrolytes—offers the added benefits of high CO_2_ solubility, negligible volatility, and strong intermediate stabilization (e.g., CO_2_^•–^).^69,70^

The unmodified Ag electrode displays a characteristic cathodic peak at −2.41 V vs Ag (Figure 5 and Table 2), consistent with the known behavior of Ag in ionic liquids in CO_2_ reduction.^71^ When decorated with TaON, this peak shifts significantly to −1.58 V, revealing a substantial decrease in the overpotential required for the reaction. Critically, the current observed in the cyclic voltammograms (CVs) recorded with TaON-modified Ag electrodes is strongly dependent on the exact CO_2_ concentration (Figure 5a,c). The lack of a reduction peak at 0 mM indicates that the cathodic reduction window is up to −2.8 V. Analysis of the CVs reveals that the peak currents are of the same order of magnitude as expected for one-electron-transfer reactions of a diffusing species in the same ionic liquid using a similar experimental setup. Increasing the amount of CO_2_ increases the peak current, while the peak-width (Δ*E*_p_) remains approximately 145 mV (at 0.1 V s^–1^), indicating that CO_2_ exhibits slow electron-transfer kinetics. By fitting the experimental data (I_pc_ vs *C*_CO_2__) to this equation, rapid access to the diffusion-coefficient value is possible and/or the active surface area of the electrode. From these data, it is evident that the presence of TaON enhances the catalytic properties of the Ag electrode toward the electrochemical reduction of CO_2_. Additional evidence for such enhancement comes from the positive shift in the cathodic peak potential (*E*_pc_) with respect to the values obtained for pure Ag under the same electrochemical conditions (up to approximately 0.8 V difference). These results indicate TaON effectively modifies the interfacial electron-transfer environment.

**Figure 5:**
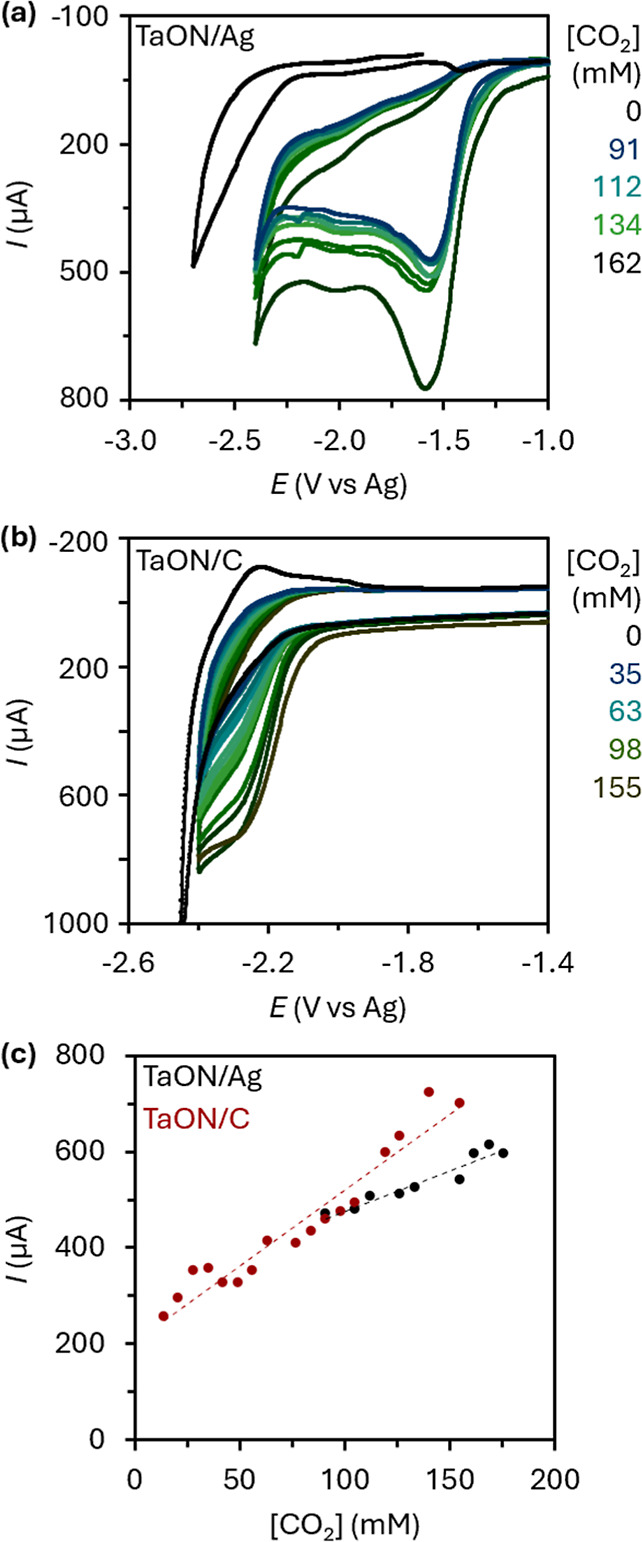
Cyclic voltammograms (scan rate 0.1 V·s^–1^, RT) recorded on TaON-modified (a) Ag and (b) C electrodes showing the electrochemical reduction of CO_2_ in the ionic liquid [N_1114_]­[TFSI] at increasing concentrations of CO_2_ (0–162 and 0–1557 mM for Ag and C, respectively). (c) Linear dependence of the cathodic peak current with CO_2_ concentration under CO_2_ flow (2 mL·min^–1^).

**Table 2:** Electrochemical Reduction of CO_2_ over TaON-Modified Electrodes^a^

electrode	*E*_pc_ (V vs Ag)
Ag	–2.41
TaON-modified Ag	–1.58
C	not detected
TaON-modified C	–2.27

a TaON was deposited at 0.1 V s^–1^.

We expect that CO_2_ reduction proceeds in a similar fashion over TaON/Ag as it does over unmodified (pure) Ag, which exhibits well-established intrinsic activity toward selective CO_2_ reduction to CO.^71–73^ In both cases, the reaction begins with an initial one-electron transfer to form the CO_2_^•–^ radical anion, which subsequently evolves to CO as the final product. The incorporation of TaON likely acts synergistically with Ag, lowering the overpotential required for CO_2_^•–^ formation and enhancing the overall electrocatalytic response toward CO_2_ reduction. In other words, the combined effect of Ag and TaON facilitates CO_2_ adsorption, activation, and subsequent reduction.

In contrast to Ag, the unmodified (pure) C electrode exhibits no discernible peak, which aligns with the low catalytic activity of C-based electrodes toward CO_2_ electroreduction.^74^ Figure 5b shows the cyclic voltammograms recorded on TaON/C electrodes over different CO_2_ concentrations (0–155 mM). Upon modification with TaON, a clearly defined cathodic peak at −2.27 V appears, indicating the catalyst activates an otherwise electrochemically inert surface (Figure 5 and Table 2). Increasing concentrations of CO_2_ result in a proportional increase in the peak current, while the peak-width value (Δ*E*_p_) remains approximately 118 mV (at 0.1 V s^–1^), characteristic of slow electron-transfer kinetics for CO_2_ reduction on the TaON-decorated carbon electrode (α = 0.41). These findings are particularly notable, given that C represents a much cheaper alternative to Ag-based electrodes. These combined results confirm that TaON enhances CO_2_ reduction on both Ag and C substrates, with more pronounced improvement with Ag due to its inherently favorable charge-transfer properties.^75^ Enhanced CO_2_ reduction with both Ag and C electrodes is consistent with the dual catalytic role proposed for metal oxynitrides—including TaON—which can promote both the adsorption and activation of small molecules while facilitating charge transfer across different conductive substrates.^76–78^

## Conclusion

Ball milling is a versatile top-down synthetic approach for accessing oxynitride nanocrystals. Both pre- and postnitridation ball milling enable the production of TaON nanocrystals with greatly improved dispersibility with respect to their bulk counterparts. Postnitridation ball milling allows for particle size control and results in darker nanocrystals with slightly different optical characteristics than those prepared through prenitridation ball milling. Remarkably, these differences have little effect on the performance of the photocatalysts in the photodegradation of the bromothymol blue. Specifically, CuO-modified Ta_3_N_5_/TaON nanocrystal heterostructures prepared through both pre- and postnitridation ball milling exhibit excellent photocatalytic activity compared to commercially available catalysts and equivalent activity to their bulk forms. This suggests that ball milling is conducive to the formation oxynitride/nitride nanocrystal heterostructures, which often exhibit more efficient charge separation. Ultimately, these results may enable the rapid adoption of TaON into large-scale flow reactors, bridging the gap between lab-scale and pilot-scale photocatalysis.

Electrochemical CO_2_ reduction in an ionic liquid medium demonstrate that TaON nanocrystals substantially enhance the CO_2_ reduction activity of screen-printed Ag and C electrodes. On silver, TaON reduces the overpotential by nearly 0.8 V, sharpens voltammetric features, and maintains high catalytic currents throughout the experiment. On carbon, TaON introduces measurable catalytic activity where none initially existed, enabling a well-defined *E*_pc_ at −2.27 V and progressively increasing cathodic currents as CO_2_ saturation increases. Present and future studies in the mechanochemical size reduction of this family of materials will play a crucial role in designing dispersible, efficient, and air stable photo- and electrocatalysts for scalable fuel production and purification processes.

## Experimental Section

### Materials

All of the materials were commercially sourced and used as received. Tantalum­(V) oxide (Ta_2_O_5_, 99.8%) was purchased from Strem; anhydrous ammonia from Airgas.

### Nitridation

Ta_2_O_5_ (350 mg, 0.8 mmol)—before or after ball milling—was added to an alumina crucible and placed in a tube furnace. Under a flow of anhydrous ammonia (20 mL/min), the sample was heated to 850 °C for 3 h, then allowed to cool to room temperature. TaON was removed from the furnace and stored in air under ambient conditions. A minor Ta_3_N_5_ impurity (14–20%^w^/_w_) was observed in samples made under a faster flow of ammonia (60 mL/min) at 800 °C for 1.5 h.

### Ball Milling

250 mg TaON (ball-to-powder ratio or BPR = 12.7) or 350 mg Ta_2_O_5_ (BPR = 9.1) was ball milled between 15 and 250 min at 1725 rpm in a hardened steel vial (0.5 in diameter × 1 in long) containing three hardened steel balls (0.25 in diameter) using an impact SPEX 8000 M mill at a frequency of 60 Hz. Specifically, the milling jar undergoes back-and-forth motion (2.25 in) combined with side-to-side movements (1 in), following a figure-8 pattern with 1060 back-and-forth cycles.

### Photocatalytic Dye Degradation

To prepare each photocatalyst, a 1:10 ^w^/_w_ mixture of CuO and semiconductor (TaON or TiO_2_) were ground together in a mortar and pestle for 5 min. The prepared photocatalyst (5 mg total, 6.3 μmol CuO, 56 μmol TiO_2_) was added to a 10^–5^ M solution (10 mL, 0.1 μmol) of bromothymol blue at pH = 8 (adjusted with NaOH). After transferring to a Schlenk flask and purging with Ar for 30 min, the solution was placed in a fan cooled Rayonet photoreactor equipped with 16 individual 420 nm lamps with a combined measured intensity of 21,900 lx. The reaction was allowed to proceed for 1 h. The solution was centrifuged, and the absorbance measured with a standard UV–vis spectrophotometer (see the Section Optical Characterization).

### Electrocatalytic CO_2_ Reduction

#### Preparation of Electrocatalyst Inks and Drop Casting Procedure

A fresh dispersion of the electrocatalyst was prepared prior to each electrode modification step. The ink was obtained by suspending 1 mg of TaON powder in 1 mL of *n*-hexane, followed by 1–2 min of sonication to ensure adequate homogenization. Subsequently, 50 μL of the dispersion was drop cast onto commercial DropSens screen-printed electrodes (Ag or C working electrodes, Ø = 3 mm), ensuring that the deposited droplet fully covered the active surface, and the electrodes were left to dry at room temperature until complete solvent evaporation.

#### Electrode Conditioning and Electrochemical Measurements

Electrochemical activation and testing were performed using butyl methylammonium bis­(trifluoromethanesulfonyl)­imide ionic liquid ([N_1114_]­[TFSI]) as the electrolyte. A total volume of 9 mL of ionic liquid was added to the electrochemical cell. The screen-printed electrodes were mounted in a custom three electrode cell configuration for all measurements. These screen-printed electrodes incorporate a 0.1256 cm^2^ Ag or C working electrode (WE), a C counter electrode (CE), and an Ag quasi reference electrode (QRE).

During the CO_2_-involving experiments, the gas flow was continuously controlled and monitored using a Bronkhorst mass-flow controller operating at a flow rate of 2 mL/min.^71,72^ These conditions were used to investigate the interaction between CO_2_ and the active surface of Ag- or C-based electrodes doped with the electrocatalyst. To ensure the precise regulation of the gaseous environment, a modular thermal massflow meter (EL-FLOW Mass Flow Meter/Controller, Bronkhorst Hi-Tec) equipped with integrated control valves was also employed. This system enabled accurate measurement and adjustment of CO_2_ flow rates within the 0.2–10 mL/min range, ensuring stable and well-defined gas delivery throughout all cyclic voltammetry experiments. All experiments were performed using a CHInstrument 660E controlled by CHI660E software. Cyclic voltammetry (CV) was recorded at a scan rate of 0.1 V s^–1^.

#### SEM and EDX Characterization of TaON-Modified Screen-Printed Electrodes

The morphology and elemental composition of the TaON electrocatalyst deposited on screen-printed Ag and C electrodes were examined with scanning electron microscopy (Zeiss EVO SEM). Representative SEM micrographs of the Ag and C substrates after drop casting show the presence of dispersed bright particles on both surfaces, consistent with the successful deposition of Ta containing grains (Figure S14). The Ag electrode exhibits the characteristic compact granular texture of screen-printed silver inks, whereas the C electrode displays a rougher and more heterogeneous graphite-based morphology. In both cases, the catalyst grains are clearly distinguishable from the underlying substrate. To confirm their chemical composition, energy-dispersive X-ray spectroscopy (EDX) analysis was performed on individual particles observed on the electrode surface. The spectrum acquired on a selected grain reveals the presence of Ta, O, and N, with atomic fractions of 29% Ta, 37% O, and 34% N, respectively. These values are consistent with the stoichiometry expected for tantalum oxynitride (TaON), confirming that the deposited particles correspond to the intended electrocatalyst. The combined SEM–EDX analysis therefore verifies both the physical presence and the chemical identity of TaON on the surface of the Ag and C screen-printed electrodes following the drop casting procedure.

### Structural and Elemental Characterization

All powder X-ray diffraction patterns were collected in a Rigaku Ultima IV diffractometer (40 kV, 44 mA) with Cu Kα radiation (λ = 1.54 Å) with a zero-background quartz sample holder. Scherrer sizes and relative weight-percent phase purities were calculated using Match! phase identification software.^79^ Crystalline unit cell representations were generated with VESTA.^80^ SEM images and EDS data were collected using a JEOL JSM-IT200 SEM equipped with an X-ray detector. A JEOL 2100 scanning transmission electron microscope (TEM) was used to collect each TEM image. The TEM samples were prepared by drop casting an aqueous solution of nanocrystals onto a carbon coated 200-mesh copper grid.

### Optical Characterization

Diffuse reflectance spectra of solids were collected with an SL1 Tungsten Halogen lamp (vis-IR), SL3 Deuterium Lamp (UV), and BLACK-Comet C-SR-100 spectrometer (200–1080 nm). UV–vis absorption spectra of solutions were collected on an Agilent 8453 spectrophotometer. Band gaps were estimated using the reported Tauc method,^81^ where the x intercept of (Ahν)^r^ graphed against hν is understood to be the band gap (A = absorbance, hν = incident photon energy in eV, *r* = 1/2 (indirect band gap semiconductors) or 2 (direct band gap semiconductors)). Dynamic light scattering (DLS) and zeta potential measurements were performed on a Malvern Zetasizer Nano ZS. Prior to DLS, solids were suspended in ultrahigh purity water and passed through a 100 nm syringe filter to remove large aggregates known to skew DLS size distributions.

## Supplementary Material



## Notes

DLS, SEM–EDS, sedimentation studies, powder XRD, diffuse reflectance, photoluminescence, and photocatalysis results (PDF)
