# Supplementary material for: Ball Mill Beatdown: Mechanochemical Synthesis of TaON Nanocrystals
Source: Inorg Chem. 2026 May 15;65(21):11827–35. doi: 10.1021/acs.inorgchem.6c00957 (PMC13231422; doi:10.1021/acs.inorgchem.6c00957)
Supplement: Supplementary file 1 [file ic6c00957_si_001.pdf]

Supporting Information

Ball-Mill Beatdown: Mechanochemical Synthesis of TaON Nanocrystals

Eve K Stegner,[1] Madison R Kuns,[1] Kerly Ochoa-Romero,[2] Gonzalo Guirado,[,2] and Javier Vela*[,1,3]*

[1]Department of Chemistry, Iowa State University, Ames, Iowa 50011, United States;

[2]Departament de Química, Universitat Autonòma de Barcelona, Cerdanyola del Vallès, 08193 Barcelona, Spain;

[3]US DOE Ames National Laboratory, Ames, Iowa 50011, United States

vela@iastate.edu

Gonzalo.Guirado@uab.cat

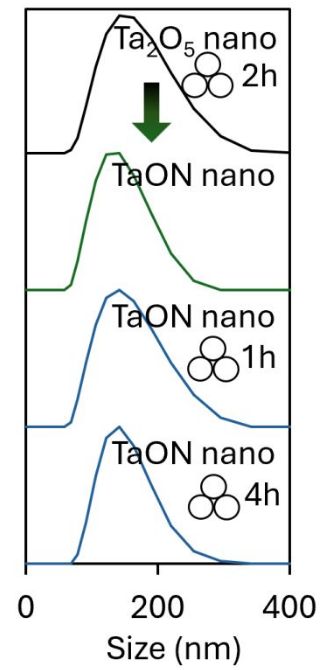

Figure S1. Dynamic light scattering (DLS) size distributions of ball-milled Ta_2O_5 (black), TaON prepared by pre-nitridation ball-milling (green), and TaON prepared by post-nitridation ball-milling (blue).

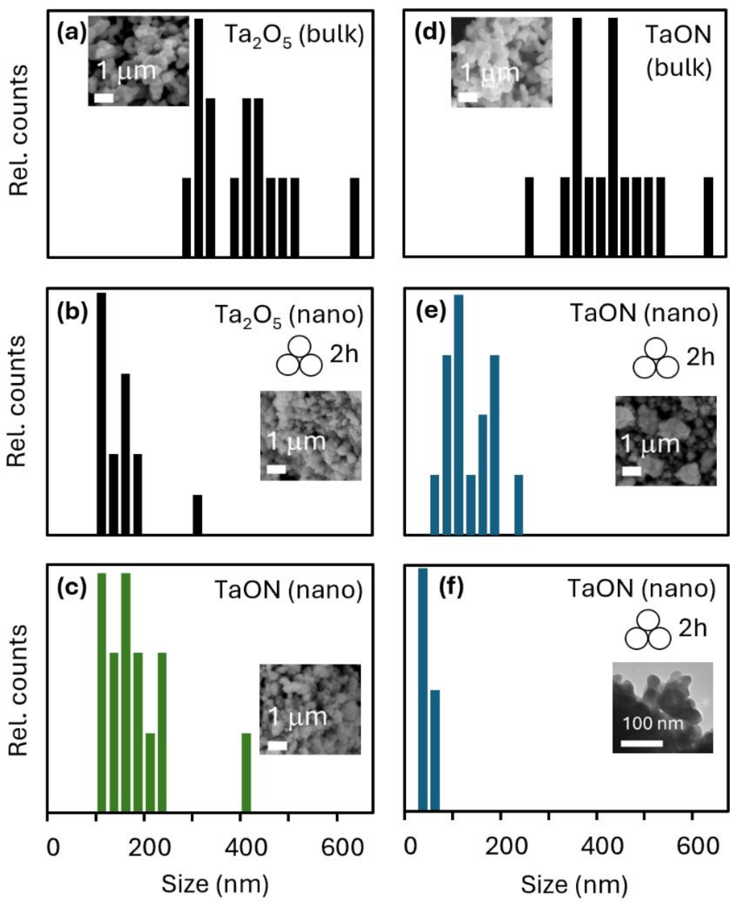

Figure S2. Size distributions obtained from (a-e) SEM and (f) TEM. TaON nanocrystals prepared from pre-nitridation ball milling are represented in green while TaON nanocrystals prepared from post-nitridation ball milling are represented in blue.

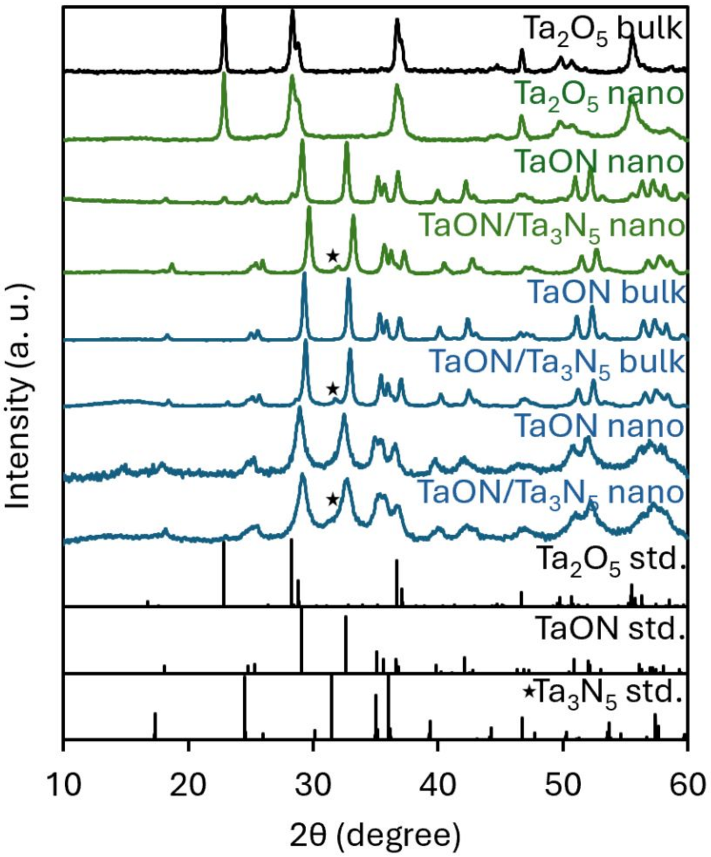

Figure S3. Powder XRD (a) of Ta_2O_5, TaON, and TaON/Ta_3N_5 (Ta_3N_5 reflection (023) indicated with black star) nanocrystals along with resulting particle sizes (b–c) from pre-nitridation ball milling (green) and post-nitridation ball milling (blue) (Ta_2O_5 *P*2*mm* #9112; TaON $P2_1/c$ #1032; Ta_3N_5 *Cmcm* #66533).

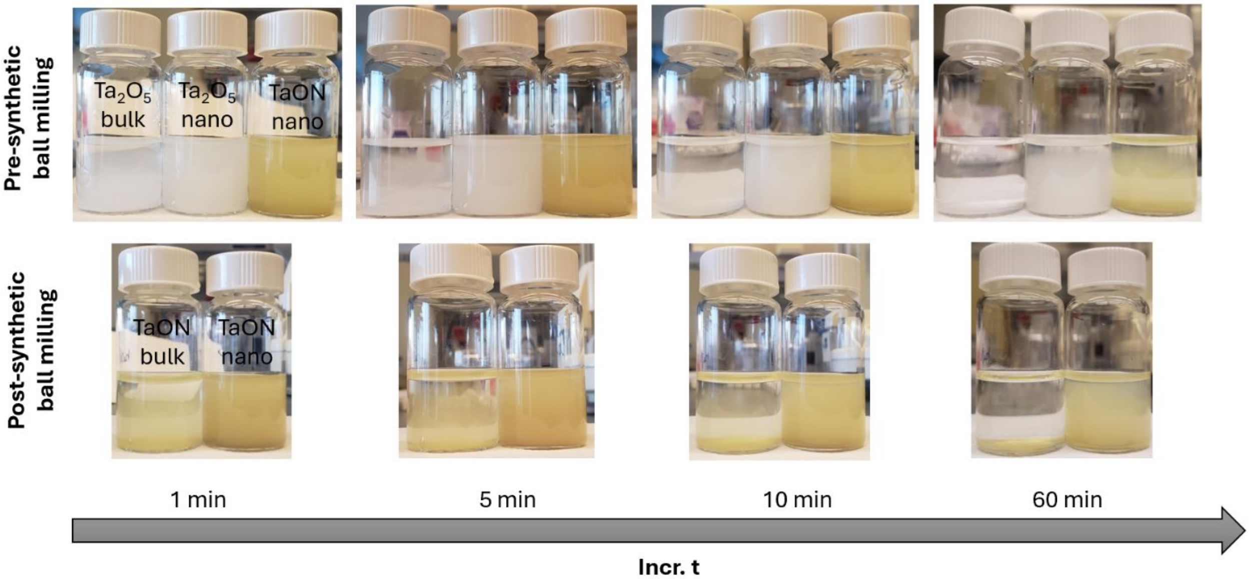

Figure S4. Sedimentation of Ta_2O_5 and TaON nanocrystals prepared from pre- and post-nitridation ball milling compared to bulk Ta_2O_5 and TaON over the course of 1 h.

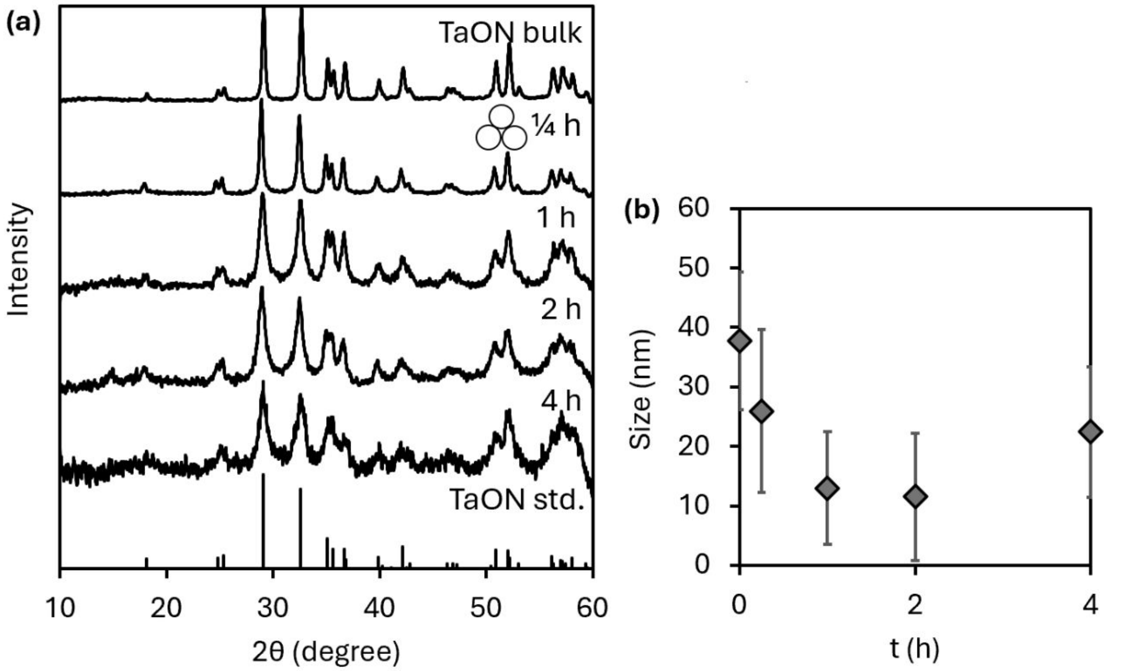

Figure S5. Powder XRD (a) and Scherrer size (b) of TaON ball milled from 0 to 4 h.

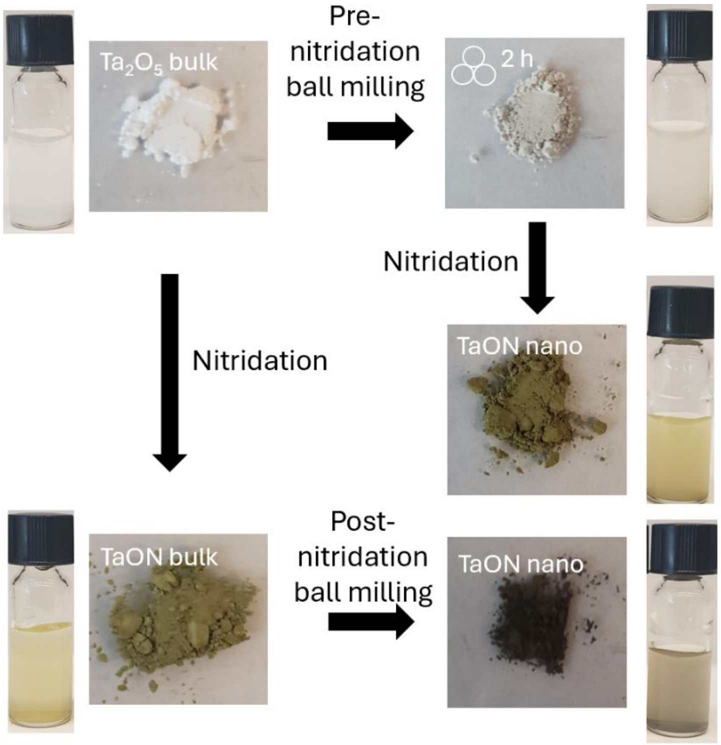

Figure S6. Color difference between TaON nanocrystals prepared from pre- and post-nitridation ball milling.

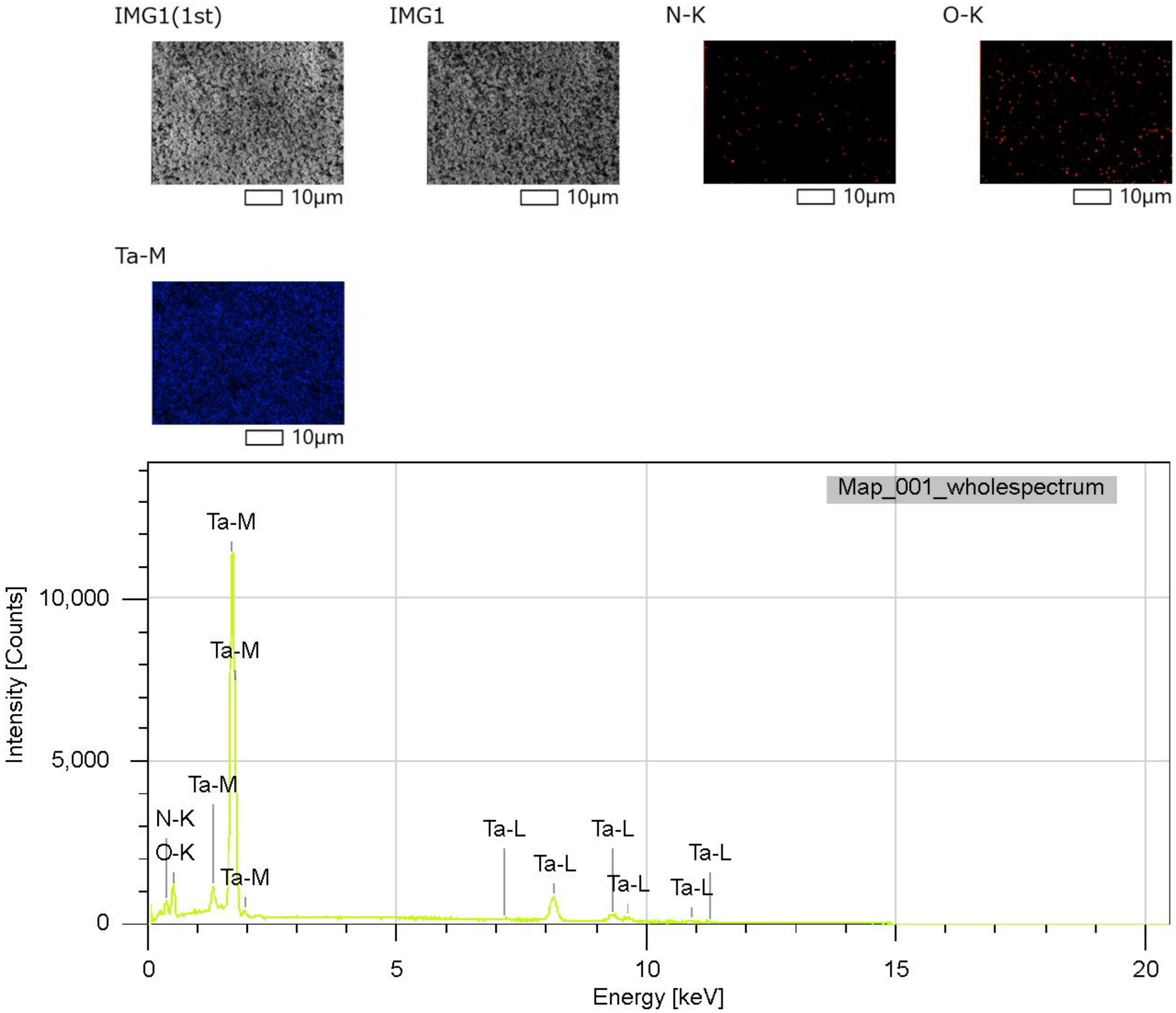

Figure S7. SEM-EDS results from post-synthetic ball milling of TaON.

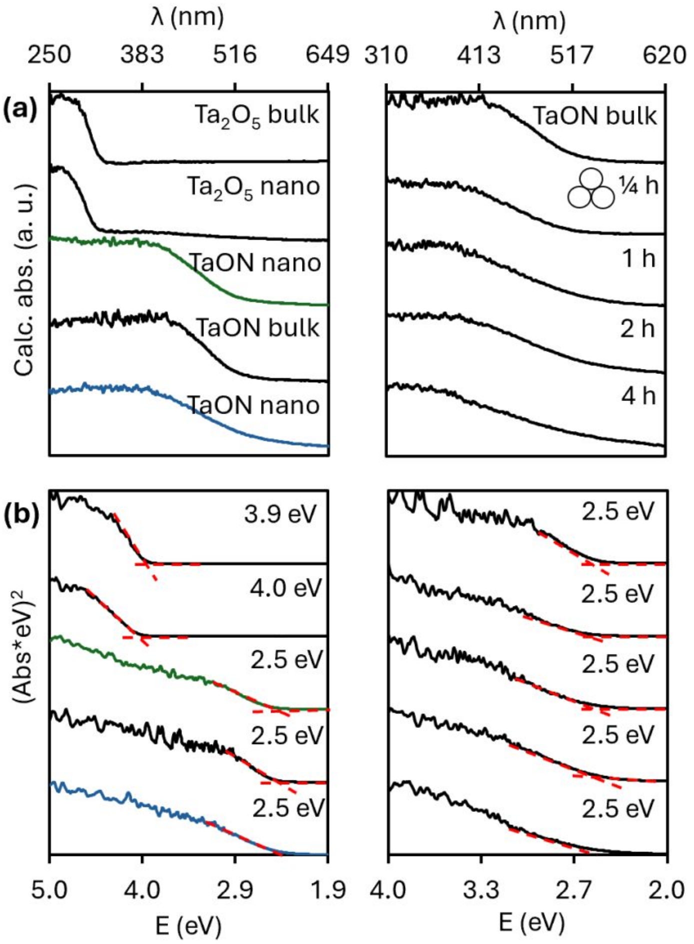

Figure S8. Leftmost graphs display absorbance spectra (a) and Tauc plots (b) of TaON nanocrystals prepared through pre- (green) and post- (in blue) nitridation ball milling. Rightmost graphs display absorption spectra (a) and Tauc plots (b) of TaON ball milled between 0 h and 4 h.

Table S1. Estimation of Bohr radii in tantalum (oxy)nitride semiconductors.[S1]

Semiconductor	ε	m_h^* / kg	m_e^* / kg	m^* / kg	a_B / nm
TaON	25.8	9.66×10^{-31}	2.82×10^{-31}	2.18×10^{-31}	5.7
Ta_3N_5	42.9	1.54×10^{-30}	9.57×10^{-31}	5.90×10^{-31}	3.5

$m = 9.11 \times 10^{-31}$ kg; $a_0 = 0.0529$ nm

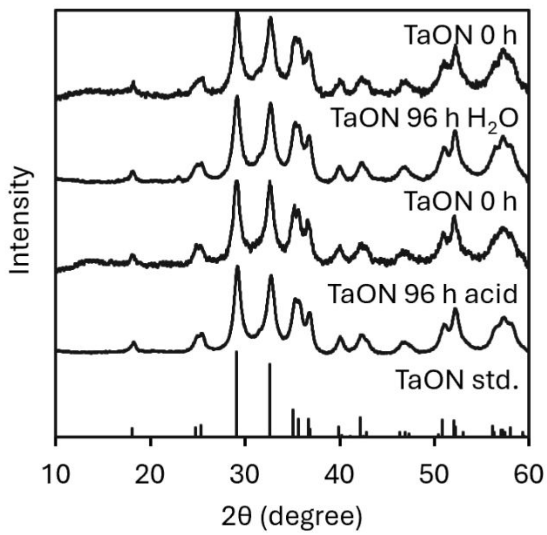

Figure S9. Powder XRD of TaON suspended in water and acid (HCl, pH = 1) for 96 h.

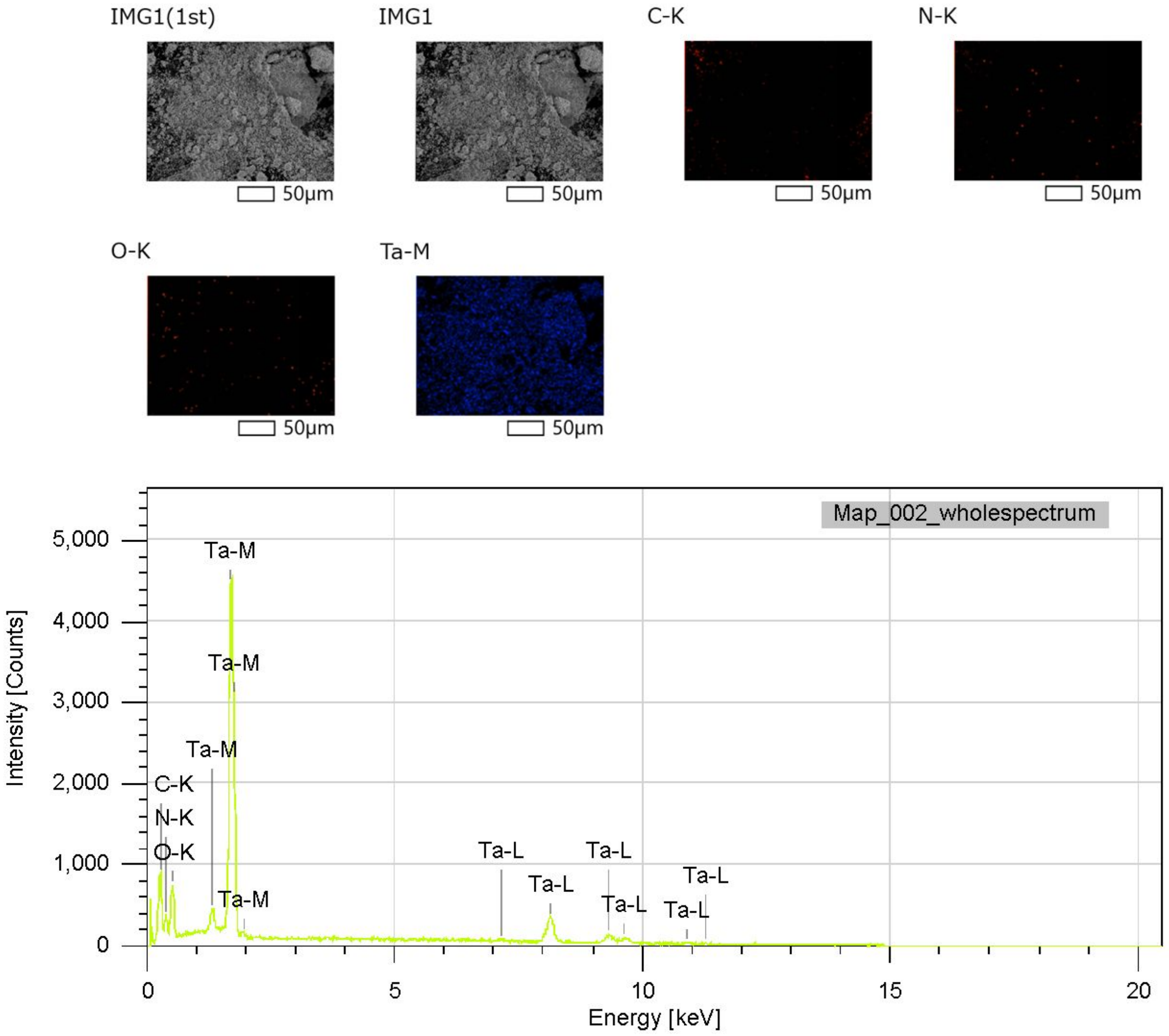

Figure S10. SEM-EDS of TaON nanocrystals (containing 20 weight percent Ta_3N_5) prepared from post-nitridation ball milling.

Table S2. Composition of TaON and TaON/Ta_3N_5 prepared from post-nitridation ball milling.

Material	EDS composition (parameterized to 1 Ta)	N/Ta
TaON	$Ta_1O_{0.8}N_{0.6}$	0.6
TaON/Ta_3N_5	$Ta_1O_{1.3}N_{1.2}$	1.2

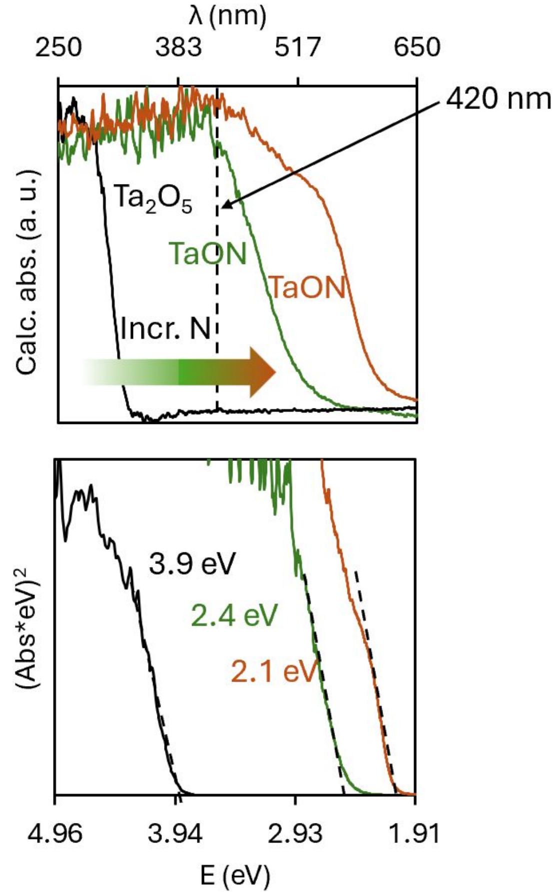

Figure S11. Calculated absorption spectra (top) and Tauc plot (bottom) of Ta_2O_5 (black), TaON (green) and TaON containing 14–20 weight percent Ta_3N_5 (orange).

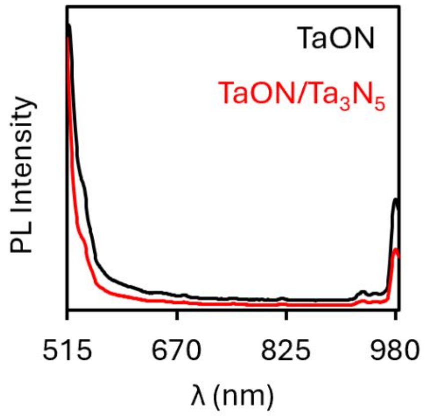

Figure S12. Photoluminescence spectra of TaON and TaON/Ta_3N_5 (slit = 5 nm, λ_{exc} = 500 nm).

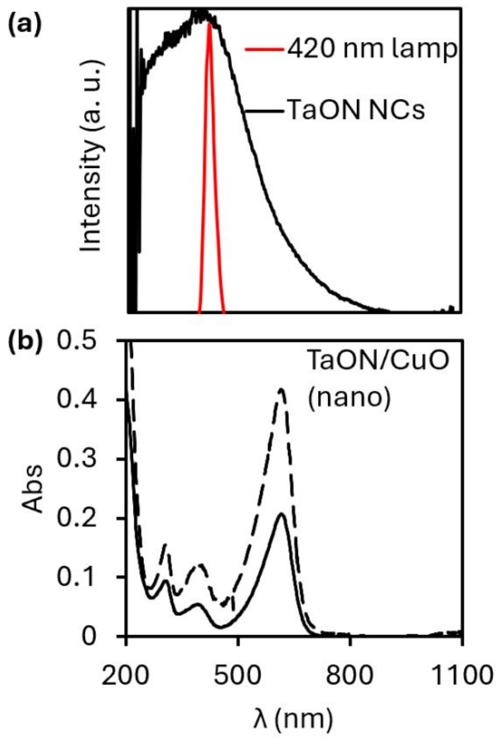

Figure S13. (a) Emission profile of 420 nm lamp in respect to TaON absorption spectrum and (b) photocatalytic degradation of bromothymol blue in the presence of TaON/CuO nanocrystals prepared from pre-nitridation ball milling.

Table S3. Bromothymol blue degradation over TiO_2 and TaON-based photocatalysts.

Semiconductor	Cocatalyst	% Degradation	AQY (%)[c]
TiO_2	CuO	23	0.002
TaON (bulk)	CuO	50	0.004
TaON (nano)[a]	CuO	52	0.005
TaON (nano) [b]	CuO	49	0.004
None	None	15	0.001

[a]Prepared from pre-nitridation ball milling. [b]Prepared from post-nitridation ball milling; t = 1 h, λ = 420 nm; 5 mg photocatalyst; 10 mL solution. [c]AQY=100×[Number of reactants consumed/ Number of incident photons (N)].

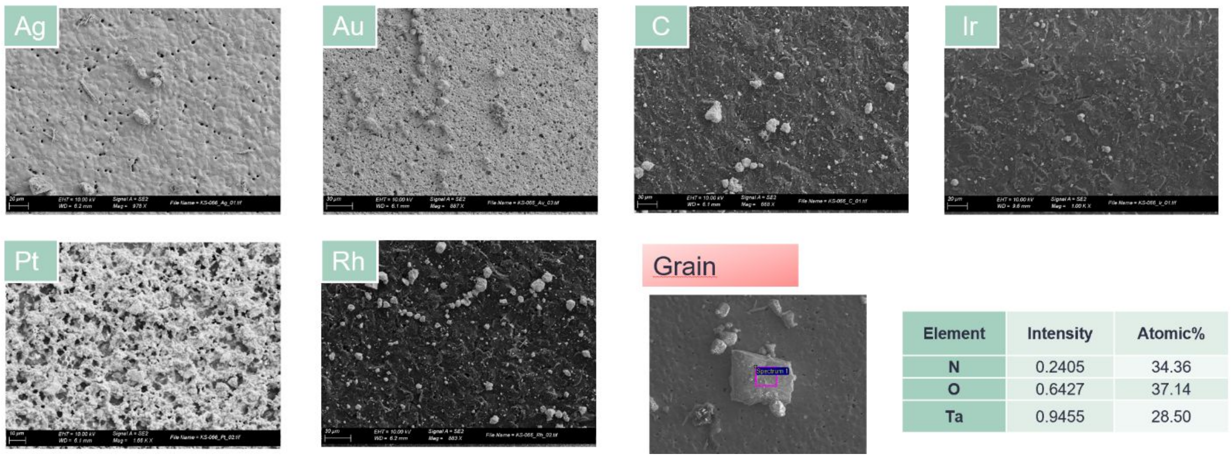

Element	Intensity	Atomic%
N	0.2405	34.36
O	0.6427	37.14
Ta	0.9455	28.50

Figure S14. SEM-EDS images and analysis of TaON coated electrodes.

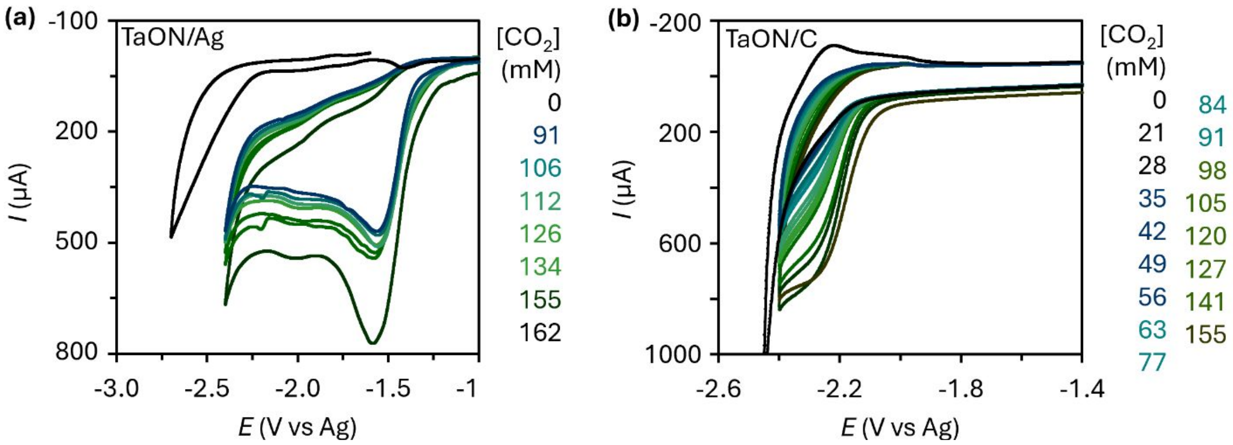

Figure S15. Cyclic voltammograms (scan rate 0.1 V·s^{-1}, RT) recorded on a TaON-modified working Ag (a) and C (b) electrodes showing the baseline and the electrochemical reduction of CO_2 at increasing concentrations in the ionic liquid [N_{1114}][TFSI]. CO_2 concentration ranges from 0–162 mM and 0–1557 mM for silver and carbon, respectively.

[S1] Nurlaela, E.; Harb, M.; Gobbo, S.; Vashishta, M.; Takanabe, K. Combined Experimental and Theoretical Assessments of the Lattice Dynamics and Optoelectronics of TaON and Ta_3N_5. *J. Solid State Chem.* **2015**, *229*, 219–227.
